# *Malat-1-*PRC2-EZH1 interaction supports adaptive oxidative stress dependent epigenome remodeling in skeletal myotubes

**DOI:** 10.1038/s41419-021-04082-z

**Published:** 2021-09-16

**Authors:** Nadine Hosny El Said, Francesco Della Valle, Peng Liu, Andreu Paytuví-Gallart, Sabir Adroub, Juliette Gimenez, Valerio Orlando

**Affiliations:** 1 Biological Environmental Science and Engineering Division, KAUST Environmental Epigenetics Program, King Abdullah University of Science and Technology (KAUST), 4700 KAUST, Thuwal, 23955-6900 Saudi Arabia; 2 Sequentia Biotech, Carrer Comte D’Urgell 240, Barcelona, 08036 Spain; 3 Epigenetics and Genome Reprogramming Laboratory, IRCCS Fondazione Santa Lucia, Rome, Italy

**Keywords:** Gene silencing, Long non-coding RNAs

## Abstract

PRC2-mediated epigenetic function involves the interaction with long non-coding RNAs (lncRNAs). Although the identity of some of these RNAs has been elucidated in the context of developmental programs, their counterparts in postmitotic adult tissue homeostasis remain uncharacterized. To this aim, we used terminally differentiated postmitotic skeletal muscle cells in which oxidative stress induces the dynamic activation of PRC2-Ezh1 through Embryonic Ectoderm Develpment (EED) shuttling to the nucleus. We identify lncRNA *Malat-1* as a necessary partner for PRC2-Ezh1-dependent response to oxidative stress. We show that in this pathway, PRC2-EZH1 dynamic assembly, and in turn stress induced skeletal muscle targeted genes repression, depends specifically on *Malat-1*. Our study reports about PRC2–RNA interactions in the physiological context of adaptive oxidative stress response and identifies the first lncRNA involved in PRC2-Ezh1 function.

## Introduction

Interaction between PRC2 and long non-coding RNA (lncRNA) is required to guarantee either proper recruitment of PRC2 at target loci or its catalytic activity [1–5]. lncRNAs such as *Xist*, *Hotair*, *Kcnq1ot1*, *Anril*, *Malat-1*, and *Terra*, regulate PRC2-EZH2 activity mediating X-chromosome inactivation, homeotic and imprinted gene silencing, and telomere length, respectively [6–11]. Other studies demonstrated that interaction of lncRNAs with PRC2 inhibits PRC2-EZH2 function through protein complex eviction from chromatin or inhibition of its catalytic activity, indicating the dual, dynamic nature of PRC2–RNA interaction [12–18].

Two variants of PRC2 are present in mammalian cells: (i) PRC2-EZH2, which is predominantly present in differentiating cells, and (ii) PRC2-EZH1, which predominates in postmitotic tissues [19–25]. Although PRC2-EZH2 controls canonical H3K27m3-dependent mitotic cell memory, the function of PRC2-Ezh1 is involved in adaptive cell response. We previously described a molecular mechanism in which a cytoplasmic short isoform of EZH1 (EZH1β) modulates shuttling of EED into the nucleus, to facilitate PRC2-EZH1α complex assembly and H3K27me3 deposition in response to oxidative stress both in vitro and in vivo [26]. In addition, regulated abundancy of both cytoplasmic and nuclear EZH1 isoforms through ubiquitination is of importance to sustain repressive role of PRC2-EZH1 under oxidative stress condition [27]. So far, RNA moieties involved in PRC2-Ezh1 function and, in particular, in postmitotic tissues have not been identified. Here we identify *Malat-1* lncRNA as a key co-factor for the PRC2-EZH1 complex dynamics and its H3K27m3 activity in response to oxidative stress in differentiated skeletal muscle cells.

## Results

### Malat-1 stabilizes PRC2-Ezh1 in postmitotic myotubes upon oxidative stress

To determine whether and which RNAs are involved with PRC2 function in postmitotic muscle cells challenged with oxidative stress, we used formaldehyde crosslinking RNA immunoprecipitation sequencing (fRIP-seq) [28]. Given the dynamics of EED relocation from the cytoplasm to the nucleus upon oxidative stress, and the complex nature of interaction between RNAs and Polycomb subunits [12, 15, 29], we focused on EED [26, 29].

EED fRIP-seq was performed on 0.1% formaldehyde crosslinked nuclei, isolated from control and H_2_O_2_ stressed myotubes, to avoid the interference from cytoplasmic EED.

**Fig. 1:**
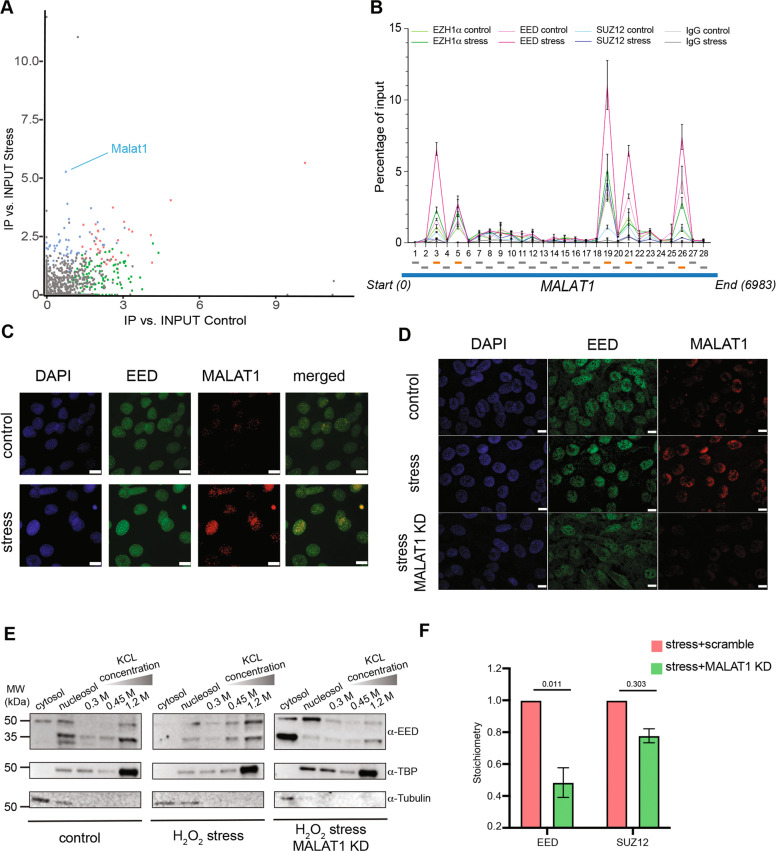
Oxidative stress-induced EED nuclear localization and PRC2-Ezh1 complex stability is supported by Malat1. **A** Scatter plot analysis of EED interacted lncRNA relative intensity in comparison with stress and control conditions. Blue, green, and red dots represent significantly enriched lncRNA bound by EED under stress condition, normal condition, and shared between stress and control conditions, respectively. Gray dots indicate background lncRNA signals. **B** CLIP-qPCR analysis to map direct interaction between MALAT1 and PRC2-EZH1a components. Relative amounts of each endogenous MALAT1 RNA regions was normalized to input for EZH1α, SUZ12, EED, and IgG using indicated color. Error bars represent ± SD from three biological replicates. Schematic illustration indicates fragments amplified in qPCR. **C** Colocalization of EED (green) and Malat1 (red) was measured using immuno-RNA FISH. DNA was counterstained with DAPI (blue). Scale bar = 10 µm. **D** Immuno-RNA FISH to detect EED (green) and MALAT1 (red), DNA was counterstained with DAPI (blue). Control and stress conditions were described as in **C**. MALAT1 KD show knockdown of MALAT using GAPMERS ASO. Scale bar = 10 µm. **E** Immunoblot analysis of EED distribution in the cytosol, nucleosol, and chromatin fractions. TBP and Tubulin were used as cytosol and chromatin fraction control. Indicated conditions have been described in **D**. **F** Stoichiometry of Ezh1a interactors EED and SUZ12 under stress conditions treated with wither scramble control or Malat1 ASO. Data are shown as mean ± SD (*n* = 3 independent tandem affinity immunoprecipitation). *P*-value shown on top of each graph using the *t*-tests (and nonparametric tests) in GraphPad Prism version 8. Relatively normalized DIA value of indicated conditions were graphed.

Strikingly, upon oxidative stress, long intergenic non-coding RNA *Malat-1* resulted to be highly enriched (Fig. 1A).

*Xist* RNA, a very well-characterized PRC2-interacting lncRNA, was used as a positive control in fRIP-quantitative PCR (qPCR) (Supplementary Fig. S1A, B).

Crosslinking immunoprecipitation and qPCR (CLIP-qPCR) analyses confirmed the direct interaction between *Malat-1* and EED at distinct regions with a lower affinity for the other PRC2 subunits EZH1α and SUZ12 in stressed cells (Fig. 1B). Immunofluorescence in situ hybridization (FISH) assay confirmed both *Malat-1* overexpression and its colocalization with EED inside myotubes nuclei treated with H_2_O_2_ (Fig. 1C). Recently, it has been shown that PRC2 complex interacts with RNA through the WD40 domains of EED and the EBD-BAM domains of EZH1/2 [29, 30].

To verify whether the interaction with *Malat-1* is functional for PRC2-EZH1 activity, we knocked down *Malat-1* using LNA Gapmers in postmitotic myotubes under H_2_O_2_ stress (Supplementary Fig. S2A). As we previously reported [26], upon oxidative stress, EED shuttles from the cytoplasm to the nucleus, to assemble a PRC2 complex and trigger H3K27 histone methyl transferase (HMT) activity. Interestingly, we found that under stress conditions, *Malat-1* depletion affected the nuclear localization of EED (Fig. 1D). In contrast, localization of SUZ12 was not affected (Supplementary Fig. S2B). As a control, we knocked down lncRNA H19, a known interactor of PRC2 [31], which is also enriched in EED fRIP experiment in stressed cells. Neither EED or SUZ12 nuclear localization under stress condition were affected upon depletion of H19 (Supplementary Fig. S3A–D). These results suggest that under oxidative stress conditions, EED proper nuclear localization, but not SUZ12, depends on *Malat-1*. Further, to check EED chromatin association, we performed cell fractionation upon oxidative stress both in wild-type and *Malat-1*-depleted cells. As expected, in H_2_O_2_-treated myotubes, we observed shuttling of different EED isoforms from the cytosol to the nucleus and chromatin compartments (higher salt concentration). Consistent with immuno-FISH data, upon *Malat-1* knockdown EED isoforms 3 and 4 (50 and 45 kDa, respectively) were redistributed from the chromatin to the cytosolic compartment, confirming that *Malat-1* stabilizes the localization of EED inside the nuclear compartment (Fig. 1E). We hypothesized that the re-distribution of EED due to *Malat-1* knockdown under oxidative stress condition could alter PRC2-EZH1 complex stoichiometry, thus impairing complex formation and HMT activity. We pulled down EZH1α and performed DIA/SWATH quantitative mass spectrometry (MS) analysis to quantify the amount of EED and SUZ12 associated with EZH1α catalytic subunit. After *Malat-1* depletion, the amount of EED interacting with EZH1α dropped dramatically, while only a minor and nonsignificant reduction occurs between SUZ12 and Ezh1α (Fig. 1F). Consistently, the HMT activity of PRC2 complex was enhanced once interacting specifically with *Malat-1* RNA (Supplementary Fig. S4A).

### Malat-1 is essential for the H3K27me3 remodeling triggered by oxidative stress

**Fig. 2:**
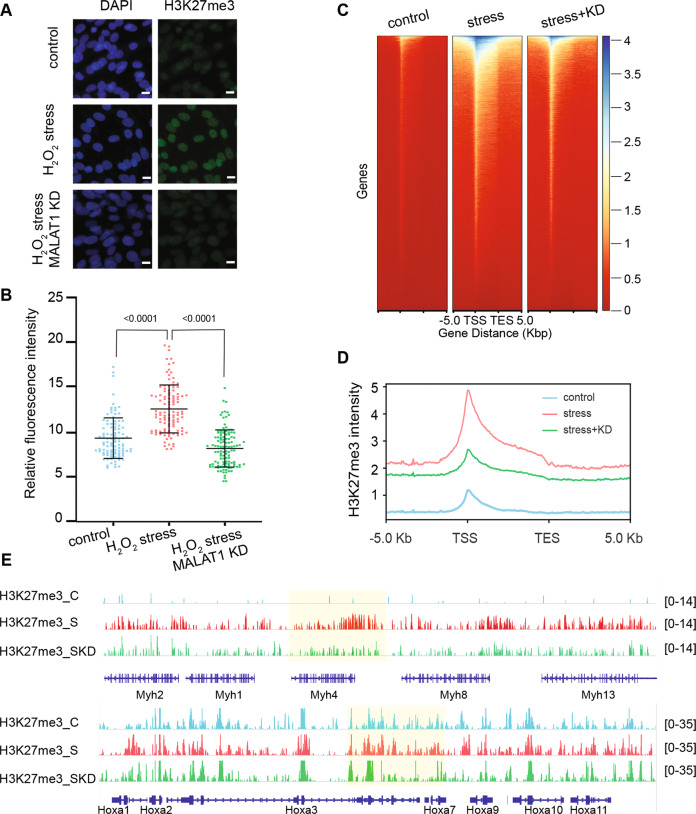
Global H3K27me3 stress-induced enrichment is compromised by *Malat-1* depletion. **A**, **B** Immunostaining of H3K27me3 (green) under indicated condition, nuclei are stained with DAPI (blue), scale bar = 10 μm (**A**). Relative H3K27me3 fluorescence intensity was graphed (**B**). Minimum 100 nuclei were counted for statistical analysis. *P*-value shown on top of each graph using the *t*-tests. **C**, **D** ChIP-seq analysis of H3K27me3 landscape under normal, stress, and stress upon Malat1 depletion conidiations. Heatmap (**C**) and metagene profiles (**D**) of H3K27me3 abundancy centered at gene body loci (±5 kb) for all genes under different indicated conditions. **E**, **F** IGV browser views of H3K27me3 profiles deposited around myosin heavy chain (MYH) gene clusters (**E**) and Hoxa gene clusters (**F**). All track signals have been normalized over spike-in control and input. H3K27me3_C, H3K27me3_S, and H3K27me3_SKD indicate H3K27me3 ChIP-seq signal under normal, stress, and stress with Malat1 depletion condition, respectively.

Previously, we reported a target increase of H3K27me3 levels mediated by PRC2-EZH1 in atrophic myotubes [26]. Thus, considering the effects of *Malat-1* on EED distribution and PRC2 complex assembly, we asked whether H3K27me3 distribution is also affected upon *Malat-1* depletion. Immunofluorescence assay shows that in oxidative stress condition *Malat-1* depletion prevents the stress-induced increase of H3K27me3 (Fig. 2A, B). We performed H3K27me3 chromatin immunoprecipitation and sequencing (ChIP-seq) analyses in differentiated myotubes under control, H_2_O_2_-treated, and H_2_O_2_-treated *Malat-1*-depleted conditions. Spike-in internal control was added for a precise quantification of H3K27me3 global levels and distribution. In line with our previous study, oxidative stress triggers global increases of H3K27me3 levels [26]. Upon *Malat-1* depletion, the majority of stress-induced H3K27me3 signals decreased significantly (Fig. 2C, D) (Supplementary Figs. S4 and S5). We focused on regions proximal to gene body, 5 Kb upstream transcriptional start site (TSS) and downstream transcriptional termination site. Both heatmap and metagene analyses showed that most of the H3K27me3 occupancy varies within this ±5 Kb window (Fig. 2C, D). Of note, after *Malat-1* depletion, genes not involved in the late myogenesis or the oxidative stress response are not affected, reinforcing the specificity of the role of *Malat-1*-PRC2-EZH1 interaction. In fact, H3K27me3 levels drop upon *Malat-1* knockdown on the myosin heavy chain gene cluster, active in differentiated myotubes and repressed upon oxidative stress, but not on the HOX-A genes cluster (Fig. 2E). H3K27me3 ChIP-qPCR and reverse-transcription qPCR (RT-qPCR) analyses on target genes, such as MyoG, Myh3, and Myh8, confirm the ChIP-seq results (Supplementary Fig. S6A, B). This data suggests that *Malat-1* regulates the activity of PRC2-EZH1 on genes involved in late cell differentiation/maturation, which are silenced in response to stress. On the contrary, it seems to have no effect on loci previously silenced during early development, which are maintaining their epigenetic memory.

### Malat-1 and H3K27me3 co-occupancy on skeletal muscle genes induce their silencing and the onset of the atrophic phenotype

**Fig. 3:**
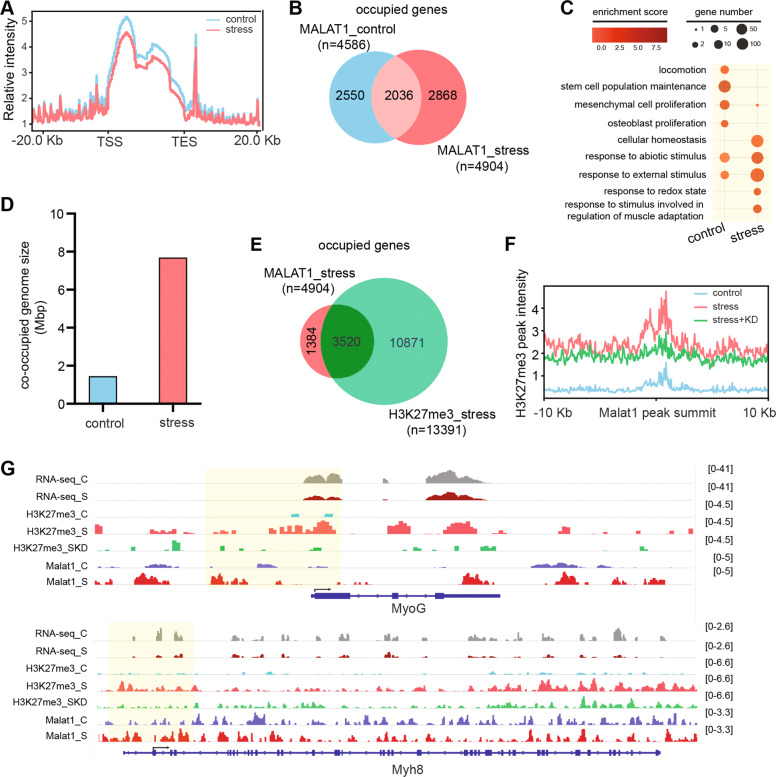
*Malat-1* and H3K27me3 co-occupy skeletal muscle-specific genes repressed upon oxidative stress. **A** Metagene plot analysis of Malat1 ChIRP-seq enrichment at centered gene bodies spanned upstream/downstream ±20 kb under normal and stress conditions. **B** Vein diagram analysis of unique and shared genes occupied by Malat1 under normal and stress conditions. **c** Enriched dot plot GO analysis of genes bound by Malat1 under normal and stress conditions. Phenotypically relevant GO terms are plotted, size of dots represent number of genes and color of dots indicate relative enrichment score. **D** Co-occupied genomic regions size with Malat1 occupancy and H3K27me3 deposition under normal and stress conditions. **e** Vein diagram analysis of Malat1-bound genes possessing H3K27me3 marks under stress condition. **F** Normalized H3K27me3 signal intensity around (±10 kb) Malat1 ChIRP-seq occupied promoter and TSS peaks under stress condition. **G** IGV browser views of RNA-seq, H3K27me3 ChIP-seq, and Malat1 ChIRP-seq profiles around Myogenin (Myog) and Myosin Heavy Chain 8 (Myh8) genomic loci under indicated biological conditions. .

Following those findings, we performed chromatin isolation by RNA purification sequencing (ChIRP-seq) to characterize global distribution of *Malat-1* under normal and oxidative stress conditions. The enrichment of glyceraldehyde 3-phosphate dehydrogenase (GAPDH) and Xist RNA was used as a control to determine *Malat-1* probe set specificity (Supplementary Fig. S7). ChIRP-seq data show that *Malat-1* is mostly binding intergenic and intronic regions (Supplementary Fig. S8A); however, we also see a significant enrichment of Malat1 on genes on the TSS (Fig. 3A and Supplementary Fig. S8B). In comparison with control and H_2_O_2_-stressed myotubes, *Malat-1* binds 2550 and 2868 different genes, respectively. Two thousand and thirty-six genes share the same *Malat-1*-binding profile in both conditions (Fig. 3B). Gene Ontology analysis shows that genes differentially bound by *Malat-1* upon stress are associated to stress response and skeletal muscle cells adaptation to environmental stimuli. (Fig. 3C).

Given the massive but locus specific effect of *Malat-1* depletion on H3K27me3 global levels, we compared H3K27me3 and *Malat-1* peaks distribution after H_2_O_2_ treatment. Intriguingly, the portion of the genome co-occupied by *Malat-1* and H3K27me3 increased from 1.45 to 7.68 Mbp upon stress condition (Fig. 3D and Supplementary Fig. S8C). Under stress, 3520 *Malat-1*-bound genes out of 4904 acquire H3K27me3 histone mark (Fig. 3E). Focusing on proximal regulatory region of these genes (proximal promoter and TSS), we find that the H3K27me3 peaks precisely overlap with the *Malat-1* peak center; this pattern was dramatically abolished upon *Malat-1* depletion (Fig. 3F). We selected two genomic loci, MyoG and Myh8, involved in late myogenesis and specifically repressed upon oxidative stress. Consistently, the enrichment of *Malat-1* on these loci under stress condition is essential for the deposition H3K27me3 on their promoter and TSS, which show high correlation with gene repression of these two genes (RNA-sequencing from ref. [26]) (Fig. 3G).

**Fig. 4:**
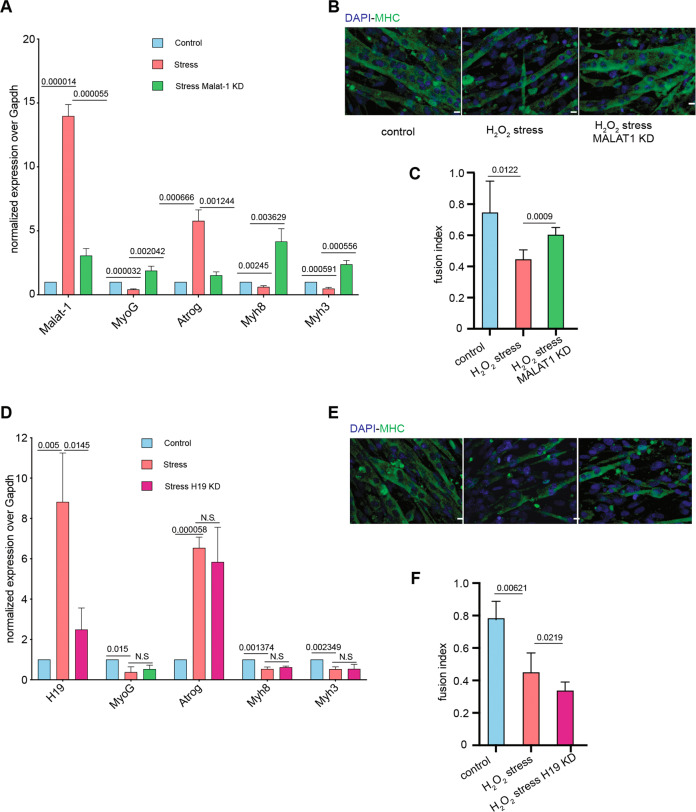
*Malat-1* depletion restores skeletal muscle gene expression and myotube fusion capacity under stress condition. **A** RT-qPCR analysis of transcription levels of *Malat-1*, *MyoG*, *Myh8*, *Myh3*, and *Atrog* under indicated conditions. Error bar represent mean ± SD (*n* = 3) with *t*-test *P*-value shown on top of each graph. **B** Immunofluorescence staining of myosin heavy chain (MYHC-green) at day 4 of differentiation. Nuclei were counterstained with DAPI (blue). **C** Fusion index analysis indicate number of nuclei in multi-nucleated myotubes, defined by at least three nuclei, divided by the total number of nuclei in the same field. Minimum four fields at ×40 magnification per replicate were counted from each experiment (~400–500 nuclei). Error bar represents mean ± SD (*n* = 3) with *t*-tests *P*-value shown on top of each graph. **D** RT-qPCR analysis of transcription levels of *H19*, *MyoG*, *Myh8*, *Myh3*, and *Atrog* under indicated conditions. Error bar represents mean ± SD (*n* = 3) with *t*-test *P*-value shown on top of each graph. **E** Immunofluorescence staining of myosin heavy chain (MYHC-green) at day 4 of differentiation. Nuclei were counterstained with DAPI (blue). **F** Fusion index analysis indicate number of nuclei in multi-nucleated myotubes, defined by at least three nuclei, divided by total number of nuclei in the same field. Minimum four fields at ×40 magnification per replicate were counted from each experiment (~400–500 nuclei). Error bar represent mean ± SD (*n* = 3) with *t*-tests *P*-value shown on top of each graph.

Of note, *Malat-1* knockdown, as a result of failure to silence myogenic genes, appeared to restore myogenic potential, by rescuing the myotubes fusion index under H_2_O_2_ treatment (Fig. 4A–C). As expected, lncRNA H19 knockdown gave no rescue of the myogenic potential of differentiated C2C12 under stress condition (Fig. 4D–F).

Recent studies characterized novel function of non-coding RNA as a bridge or structure scaffold function player to facilitate PRC2 localization to chromatin and stabilize H3K27me3 architecture [5, 32, 33]. Our study consistently proves that *Malat-1*-rich loci are prone to be silenced, corroborating the evidence of a functional correlation between *Malat-1* enrichment on chromatin, H3K27me3 deposition induced by oxidative stress, and cell adaptation.

## Discussion

In Adult tissues, cells are mostly postmitotic, continuously subjected to environmental changes and stresses during the entire lifespan. To adapt to naturally changing conditions, cells modulate their gene expression profile by regulating the activity of chromatin modifiers, including the Polycomb repressive complexes [34]. lncRNAs participate in adaptive transcriptional response, in particular during environmental stresses [29, 35, 36]. The crosstalk between RNAs and PRC2 involves multiple RNA moieties with different affinity and function [14, 15, 37, 38]. Recent findings provided compelling evidence for an essential role of RNA in stabilizing the PRC2 complex on chromatin [5]. The complex case of PRC2 may reflect a more general, still largely uncharacterized, feature of RNA–chromatin remodeler interactions. Thus, it is becoming clear that promiscuity in RNA binding is only apparent, and instead the identification of RNA–PRC2 interactions requires a systematic approach linked to specific phenotypes. Overexpression of Malat-1 is characteristic of various tumors and this has been reported to impact PRC2-Ezh2 regulation In particular, overexpression of Malat-1 is characteristic of various tumors and this has been reported to impact PRC2-Ezh2 regulation, in turn deregulated in cancer cells [39–42].

**Fig. 5:**
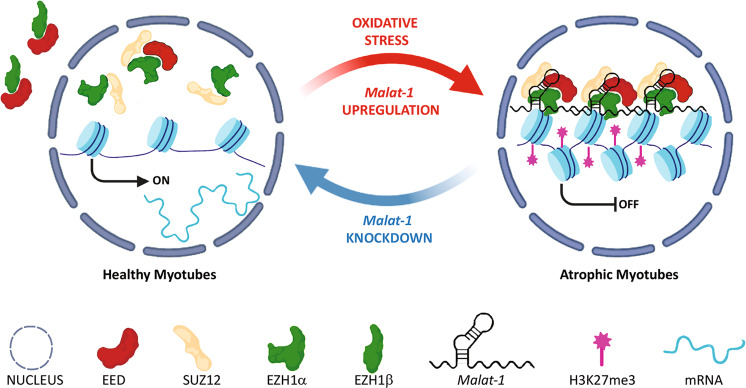
Graphical summary. Upon oxidative stress, the cytosolic fraction of EED moves into the nucleus where the upregulated Malat-1 stabilizes the PRC2-EZH1 complex on skeletal muscle genes, mediating their silencing and the onset of the atrophic phenotype. Malat-1 depletion produces a loss of H3K27me3 on myogenic genes and reverts the atrophic phenotype.

Our study adds to this complex picture, by analyzing the case of PRC2 and adaptive stress response [26, 27]. Upon oxidative stress, *Malat-1* RNA levels increase, becoming a strong interactor of EED as shown by eCLIP and fRIP data. Although future work will be required to elucidate the fine mechanistic details, we propose that for repressive PRC2-EZH1 function, *Malat-1* RNA would act as a PRC2 “attractor,” and not inhibitory component as previously described [15], allowing signal-dependent adaptive gene silencing (Fig. 5). Finally, the interaction between PRC2-EZH1 and *Malat-1* features the importance of the plastic, RNA mediated nature of PRC2 cell memory system in adult tissues, controlling stress-induced genetic program switch, a key aspect of epigenome function in maintaining homeostasis.

## Materials and methods

### Cell lines and cell cultures

Wild-type C2C12 mouse skeletal muscle cells (ATCC, CRL-1772) were grown in Dulbecco’s Modified Eagle’s medium (DMEM) (4.5 g/L d-glucose)/Glutamax (GIBCO) and 10% fetal bovine serum (FBS, GIBCO) with penicillin/streptomycin supplement. To induce differentiation, C2C12 myoblasts at 85–95% confluence media were changed to DMEM supplemented with 2% horse serum (GIBCO) with a penicillin/streptomycin supplement. To stimulate atrophy, myotubes at day 3 of differentiation were treated with 100 μM H_2_O_2_ for 24 h. All experiment were conducted on C2C12 myotubes at day 4 of differentiation. C2C12 EZH1α-Flag-HA stable cell line, previously reported [27], were cultured and differentiated like normal C2C12.

### Formaldehyde crosslinking RNA immunoprecipitation

fRIP was done following Hendrickson et al. [28]. C2C12 cells were collected by spinning for 5 min at 500 RCF, followed by washing using room temperature 1× phosphate-buffered saline (PBS). Each 5 × 10^6^ cells were resuspended in 1 ml of media without FBS or high salt (HS). We then added formaldehyde (Sigma) of a final concentration of 0.1%. Crosslinking was done for 10 min at room temperature. Quenching was performed using glycine added to a final concentration of 125 mM. The cells were spun again for 5 min at 500 RCF, then washed twice using cold PBS. The pellets were flash frozen in liquid nitrogen and stored at −80 °C for later use. The frozen pellets were then resuspended in a 1 ml RIPA lysis buffer (50 mM Tris pH 8.0, 150 mM KCl, 0.1% SDS, 1% Triton X-100, 5 mM EDTA, and 0.5% sodium deoxycholate, supplemented with freshly prepared 0.5 mM dithiothreitol (DTT), 1× EDTA protease inhibitor cocktail (Roche), and 100 U/ml RNaseOUT (Life Technologies, 10777–019)) per tube. The cells were incubated at 4 °C for 10 min, then lysed with a Branson® digital sonicator using 10% amplitude for 0.7 s ON and 1.3 s OFF at 30 s intervals for a total of 90 s. Lysate was then centrifuged at maximum speed for 10 min at 4 °C. Supernatant was collected and an equal amount of fRIP binding/wash buffer (150 mM KCl, 25 mM Tris pH 7.5, 5 mM EDTA, 0.5% NP-40, supplemented with freshly prepared 0.5 mM DTT, 1× EDTA protease inhibitor cocktail (Roche) and 100 U/ml RNaseOUT (Life Technologies, 10777–019)) were added to dilute the samples. Fifty microliters of lysate were kept out of each 1 ml as the input sample. Input samples were store at −20 °C for later RNA purification and preparation of the library. Pre-clearing was done by incubating each lysate from 5 million cells for 30 min on a rotor at 4 °C with 25 μl of Dynabeads® Protein G (Life Technologies catalog #10004D). Aliquots of 5 million cells/ml were flash frozen and stored at −80 °C for later use. To start the fRIP, the lysates were thawed and 5 μg of EED (Millipore, 03–196 | RIPAb + TM EED) or IgG (Santa Cruz, sc-2027) was added. Then the lysates plus the antibody were left to rotate at 4 °C overnight before adding the Dynabeads® Protein G. The Dynabeads were added at a concentration of 50 μl/1 ml of lysate for 1 h, to recover immunoprecipitated complexes. Washes were carried out twice using the 1 ml of fRIP binding/washing buffer. After the final wash, the beads were removed and stored at −20 °C.

### Crosslinking immunoprecipitation and qPCR

CLIP-qPCR was performed following previously described protocol [43]. Briefly, 2 million, both normal and H_2_O_2_ treatment C2C12 day 4 myotube, cells were ultraviolet (UV)-crosslinked (254 nM UV-C) at 0.4 J/cm^2^ using UVP crosslinker and then lysed in NP-40 lysis buffer (20 mM Tris-HCl at pH 7.5, 100 mM KCl, 5 mM MgCl_2_, and 0.5% NP-40, freshly adding Protease Inhibitor cocktail and RNaseOUT) and sonicated using a Branson sonicator (Duty Cycle: 50%, Output intensity: 20%, two times). Clarified lysates were partially digested RNase T1 to 1 U/μl final concentration, incubated at 22 °C for 5 min. Lysates were incubated with EZH1α, SUZ12, and EED antibodies (Supplementary Table 1) overnight at 4 °C. Immunoprecipitated complex was recovered with Dynabeads at 4 °C for 3 h. After washing, pellets were incubated with 20 units of RNase-free DNase I in 100 μl NP-40 lysis buffer for 15 min at 37 °C. Elution was performed with 0.1% SDS and 0.5 mg/ml Proteinase K for 15 min at 55 °C. RNA extraction was performed using classical acidic phenol protocol and RT-qPCR was proceeded with oligos listed in Supplementary Table 1.

### RNA purification and library preparation

Frozen beads were resuspended in 56 μl of RNase-free water, 33 μl of 3× reverse-crosslinking buffer (3× PBS (without Mg or Ca), 6% N-lauroyl sarco-sine, 30 mM EDTA, 15 mM DTT (add fresh)), 10 μl of Proteinase K (Life Technologies, catalog #AM9516). One microliter of RNaseOUT was added to re-suspend the beads and input samples. Protein degradation and reverse-crosslinking was done by incubating the samples and input for 1 h at 42 °C, followed by a second hour at 55 °C. One milliliter of Trizol (Sigma) was then added to the samples, shaking the samples followed by adding 200 μl of chloroform, then vigorous shaking for ~15 s. The samples were then centrifuged on a microcentrifuge at 4 °C with maximum speed for 20 min. The aqueous layer was then collected and 750 μl of ethanol was added to it. Two to 3 μl GlycoBlue (Invitrogen) were added and then the samples were run over Qiagen RNeasy® min-elute column (Qiagen, catalog #74204). We performed library construction using Illumina Truseq stranded total RNA (catalog number RS-122–2201) per the vendor’s instructions. Final library was pooled and sequenced at Illumina HiSeq 4000 platform.

### Chromatin immunoprecipitation and sequencing

Cells were crosslinked at 1% formaldehyde (Sigma, catalog number F8775) for 10 min at room temperature and crosslinking process was quenched using glycine per 125 mM final concentration for 5 min at room temperature. The cells were then lysed using lysis buffer 1 (50 mM HEPES-KOH pH 7.5, 10 mM NaCl, 1 mM EDTA, 10% glycerol, 0.5% NP-40, 0.25% Triton X-100). The nuclei were then pelleted, collected, and washed using lysis buffer 2 (10 mM Tris-HCl pH 8.0, 200 mM NaCl, 1 mM EDTA, 0.5 mM EGTA). This was followed by lysis using lysis buffer 3 (10 mM Tris-HCl pH 8.0, 100 mM NaCl, 1 mM EDTA, 0.5 mM EGTA, 0.1% Na-Deoxycholate, 0.5% N-laurylsarcosine). All lysis buffers are supplemented with freshly prepared 1× EDTA-free protease inhibitor cocktail (Sigma). Sonication of the chromatin was done using BRANSON A250 (four cycles of 1.5 min at 20% of amplitude and 50% of Duty Cycle). The chromatin-shearing efficiency was then controlled on 2% agarose gel. For each immunoprecipitation reaction, 5 µg of antibodies were added to 100 µg of chromatin DNA equivalents. The protein–antibody immunocomplexes were recovered by magnetic Dynabeads (protein G, Invitrogen). Bead washes were carried out using low-salt wash buffer (LS) (0.1% SDS, 2 mM EDTA, 1% Triton X-100, 20 mM Tris-HCl pH 8.0, 150 mM NaCl) and HS wash buffer (0.1% SDS, 2 mM EDTA, 1% Triton X-100, 20 mM Tris-HCl pH 8.0, 500 mM NaCl), respectively. The beads were then resuspended in elution buffer (50 mM Tris-HCl pH 8.0, 10 mM EDTA, 1% SDS). This was followed by de-crosslinking by the addition of RNase A (0.2 mg/ml) and Proteinase K (0.2 mg/ml) to remove RNA and digest protein, respectively. The extracted ChIP-DNA (using ChIP-DNA Clean & Concentrator, Zymo) was processed for qPCR analysis and high-throughput library preparation (TruSeq ChIP Library Preparation Kit, Illumina). The ChIP libraries were purified using Ampure XP beads and were quantified using qubit dsDNA HS assay kit. List of antibodies used is provided in the Supplementary Table.

### Chromatin isolation by RNA purification and sequencing

ChIRP was done following the protocol described by Chu et al. [44]. Biotin-labeled antisense oligo probes were designed against Malat-1 lncRNA and purchased from Sigma. The following conditions were met: (1) 1 probe per 100 bp of length; (2) GC% target = 45; (3) oligonucleotide length = 20; (4) spacing length of 60–80 bp; probes were numbered and even probes were pooled together, whereas the odd pool contained all the odd-numbered probes. The pools were diluted in up to a final concentration of 100 μM. C2C12 cells were strongly fixed using 1% glutaraldehyde for 10 min at room temperature. Then, quenching was done using a final concentration of 125 mM glycine for 5 min at room temperature. The cells were pelleted, lysed, and sonicated (BIORUPTOR, 30 s ON, 30 s OFF, and high intensity) for 3–4 h until the solution became clear. The sonicated samples were centrifuged at 16,000 RCF for 10 min at 4 °C and aliquoted into 1 ml samples per tube. Ten microliters for RNA input and 10 μl for DNA input were withdrawn from the samples. All input samples were stored at −20 °C for further step. Two milliliters of hybridization buffer was added to each 1 ml of sample. One microliter of 100 pmol/μl probes per 1 ml chromatin were added to each sample, which was mixed well and incubated at 37 °C for 4 h with Thermomixer shaking. C-1 magnetic beads (Invitrogen) were washed with lysis buffer and 100 μl of beads per 100 pmol of probes were added to the samples and left for 30 min at 37 °C with shaking. After five rounds of washing, the beads were resuspended with 1 ml of wash buffer. One hundred microliters was taken for RNA isolation using Trizol out of each 1 ml sample. The remaining sample was used to isolate the DNA. qPCR was performed to measure enrichment efficiency of Malat1 pull-down and GAPDH was used as the negative control [45].

### Immuno-FISH and immunofluorescence assay

The assay was carried out according to sequential IF + FISH in Adherent Cells protocol byStellaris (https://biosearchassets.blob.core.windows.net/assets/bti_custom_stellaris_immunofluorescence_seq_protocol.pdf). Stellaris® FISH Probes specific for Mouse Malat1 labeled with CAL Fluor® Red 610 fluorophore (Catalog #VSMF-3021–5, Biosearch Technologies, Inc., Petaluma, CA) were hybridized to C2C12 myotubes following the manufacturer’s instructions. Myosin heavy chain immunofluorescence and fusion index analysis were conducted as in Bodega et al. [26].

### Myotubes nuclei fractionation

The nuclei were prepared according to Suzuki et al. [46]. The nuclei were isolated from the cytosolic fraction using ice-cold 0.1% NP-40 in PBS. Nuclei and chromatin fractions were extracted using a gradient of salt concentration. Briefly, nuclei pellet were resuspended in strip buffer (10 mM Tris-HCl pH 7.4, 1 mM EGTA, 1,5 mM KCl, 5 mM MgCl_2_, 290 mM Sucrose, 0.1% Triton X-100, 1 mM DTT) for nucleosol extraction and LS buffer (20 mM HEPES pH 7.9, 25% glycerol, 203 mM KCl, 1.5 mM MgCl_2_, 0.2 mM EDTA, 1 mM DTT, 1× Complete mini EDTA-free (Roche), medium salt buffer (405 mM KCl), and an HS concentration buffer (803 mM KCl)) to sequentially isolate different chromatin fractions.

### Protein extraction for tandem affinity purification

Cytosolic and nuclear extracts were prepared using our previous protocol [26], with minor modifications. Briefly, cells were lysed in cytosolic extraction buffer (50 mM Tris-HCl pH 8.0, 150 mM NaCl, 0.5 mM EDTA, 0.5% Triton X-100, 5% glycerol). The nuclei were collected at 1500 RCF and 4 °C, and the supernatant was stored as cytosolic extracts. The nuclei were washed three times in cytosolic extraction buffer and suspended in nuclear extraction buffer (50 mM Tris-HCl pH 8, 50 mM NaCl, 0.5 mM EDTA, 0.5% Triton X-100, 5% glycerol), sonicated (BRANSON A250 with a 3.2 mm tapered microtip; two cycles of 30 s at 20% amplitude, 50% of duty cycle). The debris was pelleted at 16,380 RCF and 4 °C, and the supernatant was used for nuclear fraction extracts. Before immunoprecipitation, the NaCl concentration was adjusted to 150 mM. Extraction buffer was supplemented with fresh 1× EDTA protease inhibitor cocktail (Roche).

For tandem affinity purification (TAP), tagged proteins were immunoprecipitated with anti-Flag M2-agarose (Sigma) and eluted with Flag peptide (0.2 mg/ml). Further affinity purification was performed with anti-hemagglutinin (HA) antibody-conjugated agarose (Pierce) and eluted with HA peptide (0.2 mg/ml). The HA and Flag peptides were prepared as 5 mg/ml stock in 50 mM Tris-Cl pH 8.5 and 150 mM buffer, then diluted in a corresponding concentration in TGEN 150 buffer (20 mM Tris at pH 7.65, 150 mM NaCl, 3 mM MgCl_2_, 0.1 mM EDTA, 10% glycerol, 0.01% NP-40). Between each step, the beads were washed in TGEN 150 buffer three times.

### Protein digestion, peptide fractionation, and liquid chromatography–MS analysis

HA peptide eluted samples from the TAP assay were diluted in 8 M urea in 0.1 M Tris-HCl, followed by protein digestion with trypsin, according to the FASP protocol [47]. After an overnight digestion, the peptides were eluted from the filters with 25 mM ammonium bicarbonate buffer. The eluted peptides were processed in the desalting step by using Sep-Pag C18 Column (waters) based on the manufacturer’s instruction.

The peptide mixture was measured on a Q Exactive HF mass spectrometer (Thermo Fisher Scientific) coupled with an UltiMate^TM^ 3000 UHPLC (Thermo Fisher Scientific). The peptides were separated using an Acclaim PepMap100 C18 column (75 μm I.D. × 25 cm, 3 μm particle sizes, 100 Å pore sizes) with a flow rate of 300 nl/min. A 75 min gradient was established using mobile phase A (0.1% Formaldehyde (FA)) and mobile phase B (0.1% FA in 80% Acetonitrile (ACN)): 5–40% B for 55 min, 5 min ramping to 90% B, 90% B for 5 min, and 2% B for 10 min column conditioning. The sample was introduced into the mass spectrometer through a Nanospray Flex (Thermo Fisher Scientific) with an electrospray potential of 1.5 kV. The ion transfer tube temperature was set at 160 °C. The Q Exactive was set to perform data acquisition in Data Dependent Acquisition (DDA) mode. A full MS scan (350–1400 *m*/*z* range) was acquired in the Orbitrap at a resolution of 60,000 (at 200 *m*/*z*) in a profile mode, a maximum ion accumulation time of 100 ms and a target value of 3 × *e*^6^. The charge state screening for precursor ion was activated. The ten most intense ions above a 2*e*^4^ threshold and carrying multiple charges were selected for fragmentation using higher energy collision dissociation (HCD). The resolution was set at 15,000. The dynamic exclusion for HCD fragmentation was 20 s. The other settings for fragment ions included a maximum ion accumulation time of 100 ms, a target value of 1 × *e*^5^, a normalized collision energy at 28%, and an isolation width of 1.8.

### MS spectral library generation and DIA data analysis

All the DDA MS data files were loaded into a Spectronaut Pulsar X (version 12, Biognosys, Switzerland) for the library generation. The protein database used in this study was a combination of the Uniprot *Mus musculus* (Mouse) and proteome (Proteome ID: UP000000589) sequence. The default settings for the database match consisted of the following parameters: full trypsin cleavage, peptide length of between 7 and 52 amino acids, and the maximum missed cleavage of two. In addition, lysine and arginine were used as special amino acids for decoy generation and N-terminal methionine was removed during the pre-processing of the protein database. All the false discovery rates (FDRs) were set at 0.01 for the peptide-spectrum match, peptide, and protein. The Biognosys default spectral library filters included amino acid length of ion of more than two, ion mass-to-charge between 300 and 1800 Da, and a minimum relative intensity of 5%. The best three to six fragments per peptide were included in the library. The iRT calibration was required with a minimum R-Square of 0.8.

DIA data analysis has been reported previously [48]. In detail, DIA data were analyzed using Spectronaut software against the spectral libraries to identify and quantify peptides and proteins. The Biognosys default settings were applied for identification as follows: excluding duplicate assay, generation decoy based on mutated method at 10% of library size, and estimation of FDRs using *Q*-value as 0.01 for both precursors and proteins. The *p*-value was calculated by a kernel-density estimator. Interference correction was activated and a minimum of three fragment ions and two precursor ions were kept for the quantification. The area of extracted ion chromatogram at MS2 level were used for quantification. The peptide (stripped sequence) quantity was measured by the mean of one to three best precursors and the protein quantity was calculated accordingly by the mean of one to three best peptides. Local normalization strategy and *q*-value sparse selection were used for cross-run normalization. The differential expression was determined by performing one sample Student’s *t*-test. Proteins with a fold-change of >1.5 and a *q*-value of <0.01 were considered as differentially expressed proteins.

### fRIP-seq bioinformatics analysis

The fRIP-seq raw sequencing data were first trimmed and clipped using the BBDuk tool (https://jgi.doe.gov/data-and-tools/bbtools/). A minimum base quality of 25 and a minimum read length of 35 nt were set. High-quality reads were then processed with Kallisto (https://pachterlab.github.io/kallisto/about.html) to perform the expression quantification of the GRCm38 transcripts. In order to identify significantly enriched transcripts, each sample was compared with the corresponding IgG sample with NOISeq (https://bioconductor.org/packages/release/bioc/html/NOISeq.html) using the TMM normalization method. A *t*-test was performed to identify differential enrichment between control and stressed samples.

### ChIP-seq data processing, peak calling, and differential binding analysis

Quality check and trimming were performed using BBDuk, setting a minimum read length of 35 bp and a minimum Phred quality score of 25; adapters and low-quality reads were removed, while preserving their longest high-quality regions. Trimmed reads were mapped to the mouse reference genome (mm10) using a BWA (version 0.7.17-r1188). For the H3K27me3 samples, reads were also mapped against the *Drosophila melanogaster* reference genome (BDGP6) due to the spike-in added. Afterwards, H3K27me3 mapping files against mouse and *D. melanogaster* were disambiguated with the Disambiguate algorithm [49]. All mapping files underwent duplicate removal with Picard (version 2.18) and only properly paired reads with mapping quality ≥ 30 were considered using SAMtools (version 1.7). BigWigs and heatmaps were created with DeepTools (version 3.2).

Peak calling was performed using MACS2 [50]. For H3K27me3 samples, MACS2 was called with the following additional arguments: “-broad,” “-broad-cutoff 0.1.” Only peaks with a *q*-value ≤ 0.05 were considered. Peaks were annotated with HOMER (version 4.9.1).

A differential binding analysis was performed on the identified peaks using diffBind R package (version 2.12). For the H3K27me3 samples, ChIPSeqSpike R package (version 1.5) was used to infer the normalization factors relative to the exogenous DNA from spike-ins. Specifically for the H3K27me3 samples, diffBind R package code was modified to properly handle the normalization factors predicted by ChIPSeqSpike.

The ChIRP-seq raw sequencing data were first trimmed and clipped using the BBDuk tool (https://jgi.doe.gov/data-and-tools/bbtools/). A minimum base quality of 25 and a minimum read length of 35 nt were set. High-quality reads were then mapped against the Ensembl *Mus musculus* reference genome (GRCm38) with STAR (https://github.com/alexdobin/STAR). For each sample, the fragment length was estimated with sam-stats (https://expressionanalysis.github.io/ea-utils/). Peak calling was then performed separately for even and odd probes with SICERpy (https://github.com/dariober/SICERpy), with the following options: effGenomeSize 0.66, redThresh 1, fragSize X, filterFlag 2180, windowSize 200, and gapSize 1. The fragSize option was changed for each sample according to the results of sam-stats. IgG samples were used as background. The SICERpy predicted peaks were filtered to keep those with an FDR ≤ 0.05 and an enrichment fold of at least 1.5. The peaks obtained from even and odd probes were merged. A functional annotation of the peaks was performed by intersecting the coordinates of the peaks with the gene coordinates with Bedtools (https://bedtools.readthedocs.io/en/latest/).

Gene Ontology Enrichment Analyses were performed with the tool AIR (https://transcriptomics.sequentiabiotech.com/). Correlation plots were produced with DeepTools (https://deeptools.readthedocs.io/en/develop/), whereas heatmaps were produced with NGSplot (https://github.com/shenlab-sinai/ngsplot). Venn diagrams were produced with Venn tools (http://bioinformatics.psb.ugent.be/webtools/Venn/).

### Statistical analysis

In bar plots, values are presented as means and SD; number of replicates are indicated in the figure legends. To determine the significance between two mean values, we made comparisons by two-tailed *t*-test. Comparisons among three or more samples were done by one-way analysis of variance. For all statistical tests, a 0.05 level of confidence was accepted for significance. All the statistical analyses were performed with GraphPad Prism8 software.

## Supplementary information

Supplementary Figures

Supplementary Table S1

## Data Availability

The methods in detail, source data, and other datasets supporting the conclusions of this study are included within the article and its additional files. All deep sequencing data reported in this paper have been submitted to the NCBI SRA (https://www.ncbi.nlm.nih.gov/sra) under the accession number PRJNA661400.

## Materials availability

All biological materials and bioinformatics analysis scripts used in this study are available from the corresponding author on reasonable request
