# Supplementary material for: Malat-1-PRC2-EZH1 interaction supports adaptive oxidative stress dependent epigenome remodeling in skeletal myotubes
Source: Cell Death Dis. 2021 Sep 16;12(10):850. doi: 10.1038/s41419-021-04082-z (PMC8445987; doi:10.1038/s41419-021-04082-z)
Supplement: Supplementary file 1 — Supplementary Figures [file 41419_2021_4082_MOESM1_ESM.pdf]

Supplementary Figure S1

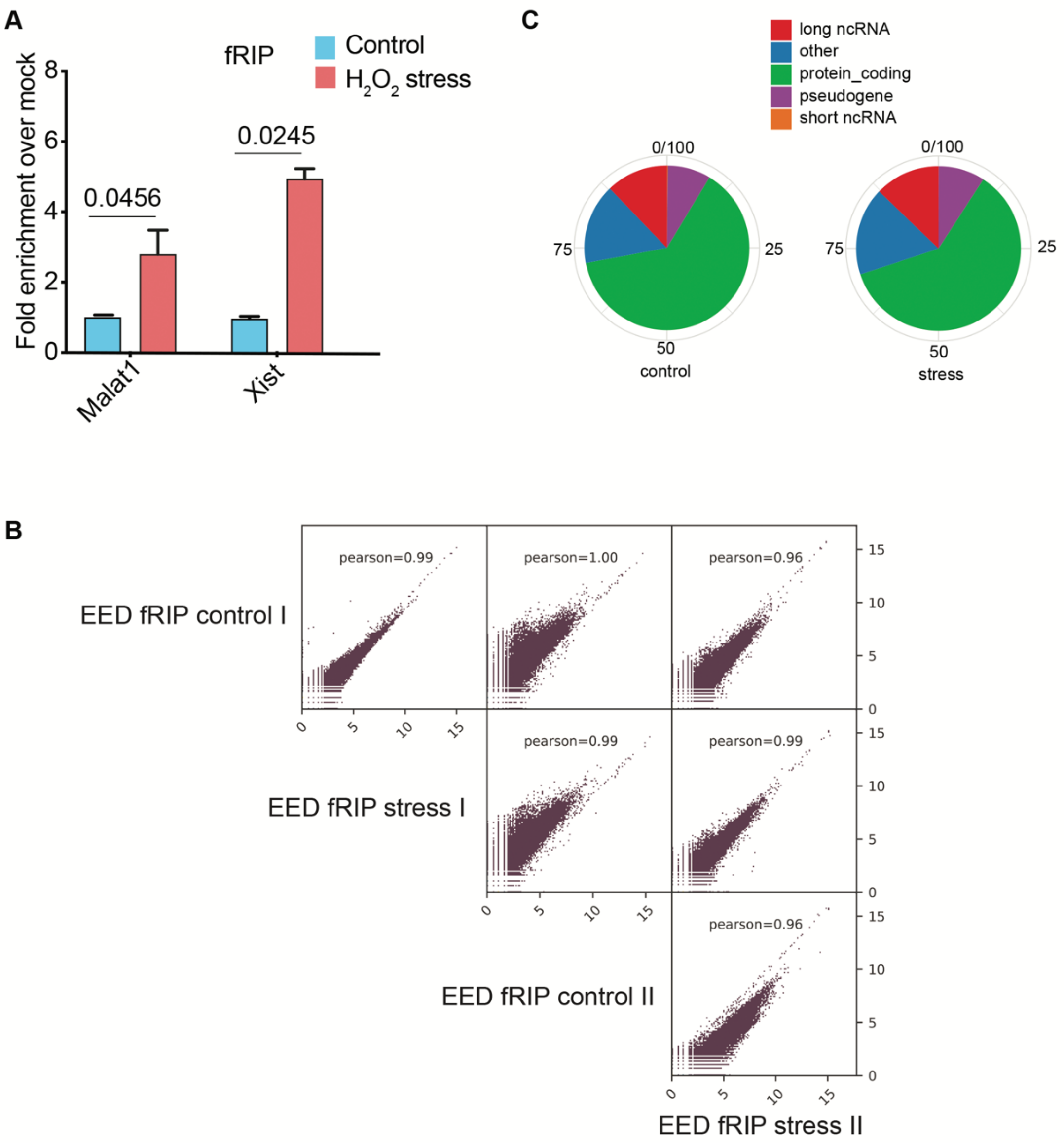

Supplementary Figure S2

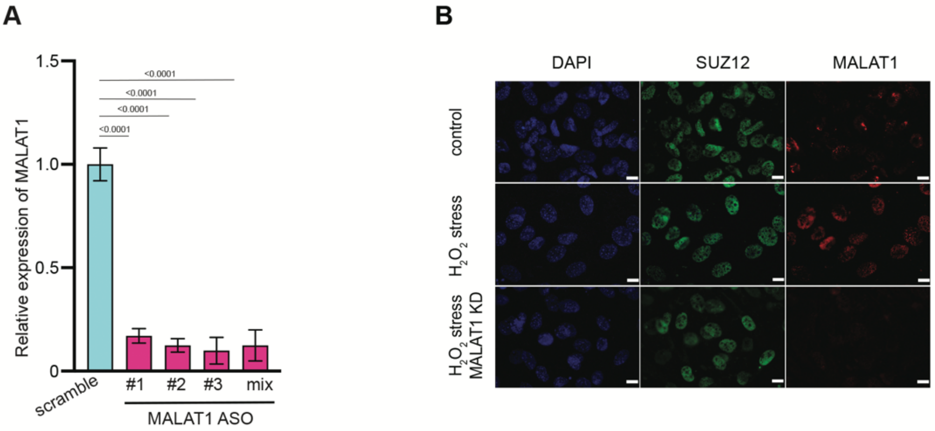

Supplementary Figure S3

A

Transcript Name	Enrichment Stress/Control	p-Value Stress/Control
2310047D07Rik-201	11.90	5.00E-01
Gm19705-204	9.80	5.37E-01
MALAT1	4.51	4.78E-01
H19	2.47	5.00E-01

B

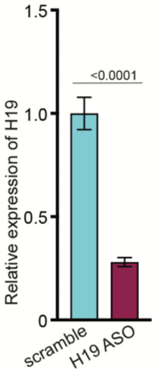

C

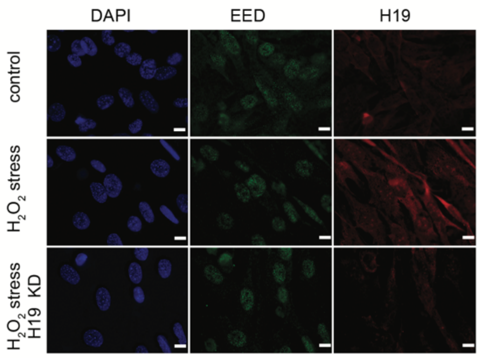

D

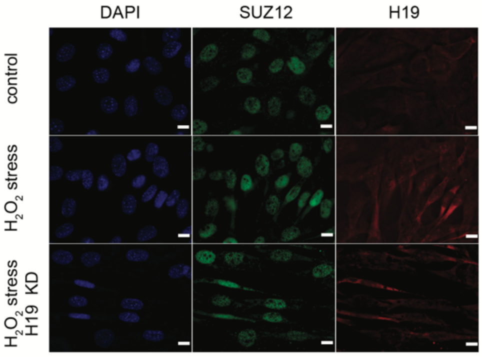

Supplementary Figure S4

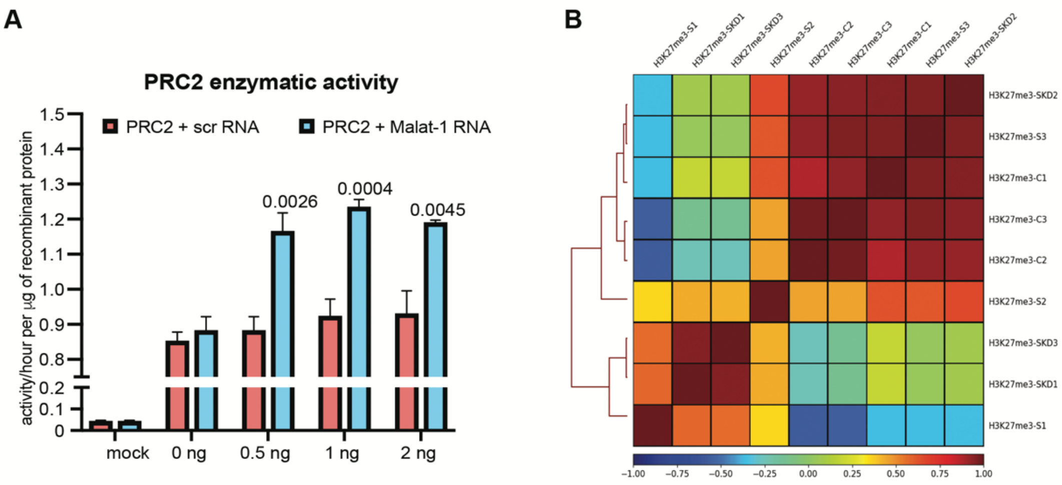

Supplementary Figure S5

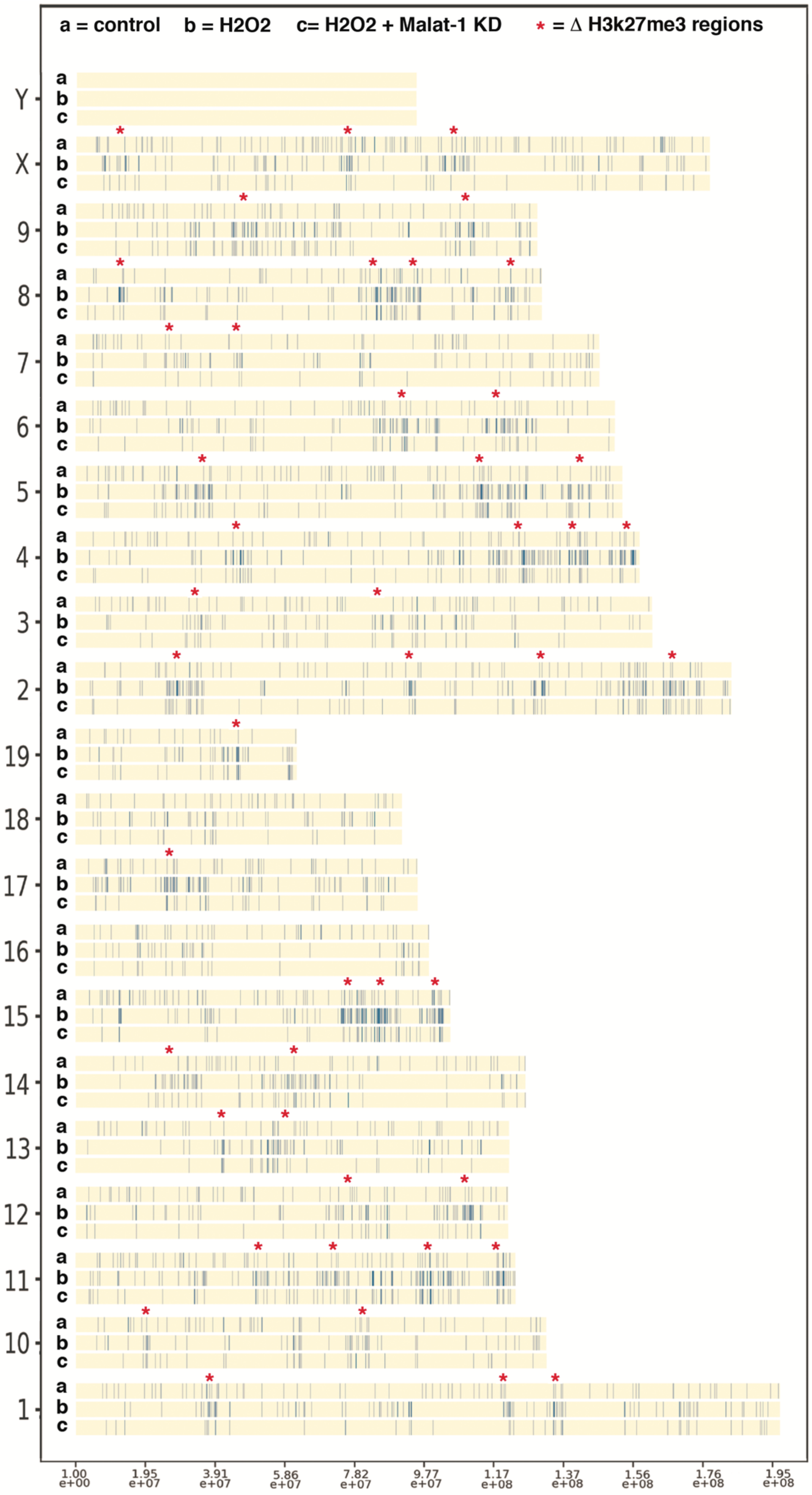

Supplementary Figure S6

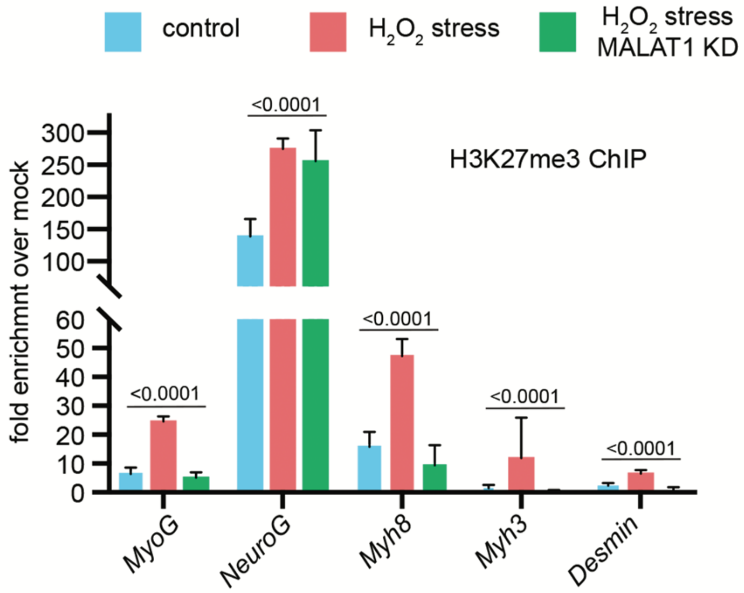

Supplementary Figure S7

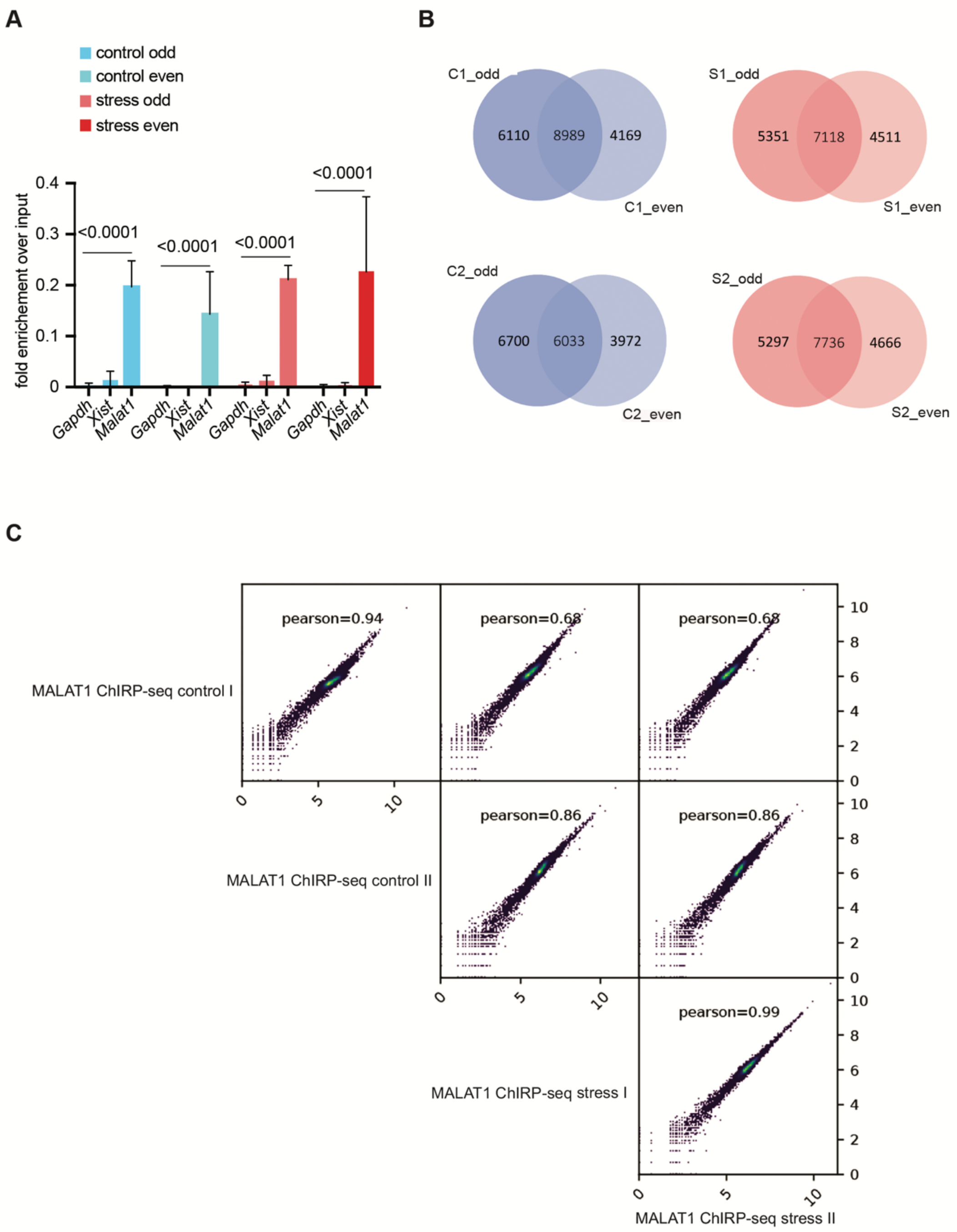

Supplementary Figure S8

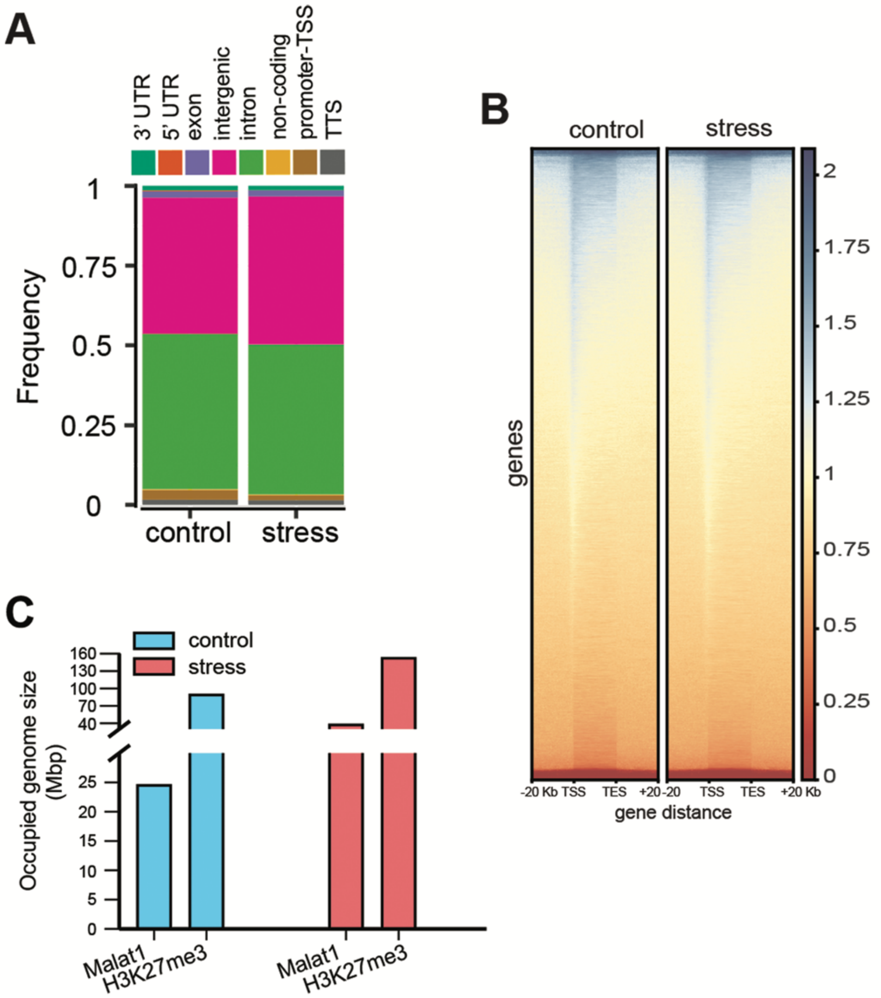

Supplementary Figure S1. Quality control and correlative analysis of different fRIP biological replicates.

A fRIP RT-qPCR analysis of fold enrichment over mock on Malat1 and Xist under normal and stress conditions. Error bar mean ± s.d. (n=3). *P* value shown on top of each graph using the *t* tests (and nonparametric tests) in GraphPad Prism version 8. **B** Scatter plots representing genome-wide correlation between different fRIP biological replicates. **C** Pie chart analysis of EED immunoprecipitated RNA species via fRIP assay, classified into protein coding RNA, lncRNA and other indicated species.

Supplementary Figure S2. Localization of SUZ12 was not affected by *Malat-1* depletion.

A RT-qPCR analysis of Malat1 expression under scramble and Malat1 ASO treatment. Error bar mean ± s.d. (n=3). *P* value shown on top of each graph using the *t* tests (and nonparametric tests) in GraphPad Prism version 8. **B** Immuno-RNA FISH to detect SUZ12 (green) and MALAT1 (red) , DNA was counterstained with DAPI (blue). Control and stress conditions were described as previously. MALAT1 KD shown knock-down of MALAT Using GAPMERS ASO. Scale bar =10μm.

Supplementary Figure S3. Effect of lncRNA H19 kncockdown on localization of EED and SUZ12.

A Differential enrichment of Malat1 and H19 in EED fRIP-seq under stress condition versus with control condition. **B** RT-qPCR analysis of H19 expression level upon scramble and H19 ASO treatment. Error bars represent mean ± s.d. (n=3), *P* value shown on top of each graph using the *t* tests (and nonparametric tests) in GraphPad Prism version 8. **C, D** Immuno-RNA FISH to detect EED (green) (**C**), SUZ12 (green) (**D**) and H19 (red), DNA was counterstained with DAPI (blue). Control, stress and stress plus H19 KD conditions were described as previously. Scale bar =10μm.

Supplementary Figure S4. Effect of Malat-1 kncockdown on PRC2 enzymatic activity.

A ELISA assay of PRC2-EZH1 Histone Methyl Transferase (HMT) activity in the presence of scramble RNA (in vitro transcribed pBluescript II SK+ plasmid) or *Malat-1* RNA**. B** Correlative analysis of different H3K27me3 ChIP-seq biological replicates under indicated conditions.

Supplementary Figure S5. Chromosome wise distribution/enrichment of H3K27me3 peaks under normal, stress and stress plus Malat1 KD treatment.
Global chromosomal distribution of H3K27me3 Spike-in normalized ChIP-seq peaks in myotubes (track "**a**"), oxidative stress treated Myotubes (track "**b**") and H2O2 treated- *Malat-1* depleted myotubes (Track "**c**").

Supplementary Figure S6. H3K27me3 enrichment on myogenesis involved genes under indicated conditions.

A ChIP-qPCR analysis of H3K27me3 status on genomic loci of *Myogenin* (*Myog)* promoter, *Myosin heavy Chain 8* and *3* (*Myh8- Myh3)*, *NeuroG* and *Desmin* under indicated conditions. ChIP enrichments are shown as relative enrichment ratio over mock. Error bars represent mean ± s.d. (n=3), *P* value shown on top of each graph using the ANOVA Brown-Forsythe test in GraphPad Prism version 8.

Supplementary Figure S7. Quality control of *Malat1* ChIRP-seq enrichment.

A RT-qPCR analyses of enrichment score over input on genes: *Malat1*, *Xist*, *Gapdh* using both *Malat1* odd and even probe pools. Error bars represent mean ± s.d. (n=3), *P* value shown on top of each graph using the ANOVA Brown-Forsythe test in GraphPad Prism version 8. **B** Venn diagram

showing the number of *Malat1*-bound peaks using both odd and even probes under control condition versus stress condition. C1 and C2 indicate two biological normal condition replicates, S1 and S2 indicate stress condition replicates. **C** Scatter plot analyses showing correlation among different *Malat1* ChIRP-seq biological replicates under both control and stress conditions.

Supplementary Figure S8. Data integration of Malat1 ChIRP-seq and H3K27me3 ChIP-seq.

A Percentage of Malat1 binding sires around the whole genome under normal and stress conditions, including intergenic, intronic and other indicated regions **B** Heatmap analysis of *Malat1* ChIRP-seq centred all genes (± 20kb) under normal and stress condition. **C** Analysis of occupied genome size bound by Malat1 and with deposited H3K27me3 histone mark under both control and stress conditions.
